# Levelling Up for health in towns? Development of a new deprivation index: the ‘Stronger Towns Index’ and its association with self-rated health and migration in England, between 2001 and 2011

**DOI:** 10.1007/s10389-023-01944-y

**Published:** 2023-06-08

**Authors:** Oliver Duke-Williams, Jemima Stockton, Nicola Shelton

**Affiliations:** 1 Department of Information Studies, University College London (UCL), London, WC1E 6BT UK; 2 Centre for Longitudinal Study Information and User Support (CeLSIUS), Department of Epidemiology and Public Health, University College London (UCL), London, WC1E 6BT UK

**Keywords:** Census, Deprivation, Geodemographic, Social sciences, Health sciences, Towns Fund, Levelling Up

## Abstract

**Aim:**

To develop the ‘Stronger Towns Index': a deprivation index that took into account characteristics of areas encompassing towns that may be eligible for redevelopment funding and explore how this index was associated with self-rated health and migration within England between 2001 and 2011.

**Subject and methods:**

All members of the ONS Longitudinal Study in England aged 16 and over in 2001 whose records included a self-rated health response and a valid local authority code.

Local authorities in England were ranked using a composite index developed using the five metrics set out in the Stronger Towns Funding: productivity, income, skills, deprivation measures, and the proportion of people living in towns.

The index was split into deciles, and logistic regression carried out on the association between decile and self-rated health in 2001 in the main sample (*n* = 407,878) and decile change and self-rated health in 2011 in a subsample also present in 2011, with migration information (*n* = 299,008).

**Results:**

There were areas in the lowest deciles of Town Strength who did not receive funding. After multiple adjustment, LS members living in areas with higher deciles were significantly more likely (7% to 38%) to report good health than those in the lowest decile in 2001. Remaining in the same decile between 2001 and 2011 was associated with 7% lower odds of good self-rated health in 2011.

**Conclusion:**

It is important to consider health in towns when allocating funding. Areas in the Midlands may have missed out on funding which might help mitigate poor health.

## Introduction

The British Government has talked recently about levelling up ‘to ensure that no community is left behind and provide associated funding.’ (MHCLG 2021). The Ministry of Housing, Communities and Local Government’s (MHCLG) announcement on the Stronger Towns Fund in 2018 suggested allocation of funding ‘based on a combination of productivity, income, skills, deprivation measures, and the proportion of people living in towns.’ (MHCLG 2019). MHCLG did not publish its methodology for calculating prioritisation of towns for funding at the outset. Subsequently, they shared some details and initially selected 541 of 1,082 towns in England above the median value income deprivation and assessed these towns further against seven core criteria: income deprivation, skills deprivation, productivity, EU Exit exposure; exposure to economic shocks, investment opportunity, and alignment to wider government intervention plans for low-priority areas (Reuben 2021). Ministers selected all 40 high-priority towns, 49 medium-priority, and 12 low-priority to apply for funding (NAO 2020). There was criticism of this selection in the media (Syal 2020), from the political opposition (Reed 2020), and from the National Audit Office. By March 2021, only 52 of 101 towns had been allocated funding (MHCLG and Jenrick 2020; BBC 2021). The funding allocation did not use the criteria they originally advertised. We were interested to see if the original criteria selection by MHCLG were associated with risk factors and outcomes that might be amenable to improvement with the addition of relatively small amounts of financial support. This could have justified their retention and use as criteria.

A fuller policy was set out in the government's White Paper 'Levelling Up' (HM Government 2022), which had a stated aim of reducing geographical disparities based on a mixture of new and existing policies (Harari and Ward 2022). The paper established four objectives, to: (1) boost productivity, pay and jobs, (2) spread opportunities and improve public services, (3) restore a sense of community, local pride and belonging and (4) empower local leaders and communities. Doubts were raised over the viability of plans for implementation of these objectives (Marmot 2022; Institute for Government 2022).

The amenability of each of the five original domains (productivity, income, skills, deprivation measures and the proportion of people living in towns) to improvement with additional financial support was considered. Income and skill levels are likely to change through longer term investments targeted at job creation and in education; both might also change through internal migration. Migration might also reflect changes in urban life due to the COVID-19 pandemic. Deprivation was measured using the Index of Multiple Deprivation (IMD) 2015 (Smith et al. 2015; MHCLG 2018), some components of which may be more amenable to change with relatively small place-based investment than others. In order to appraise interventions to reduce inequalities, a suitable framework of evidence is required (Davey et al. 2022). Murray et al. (2022) found the strongest association with not being in work in England and Wales was with self-rated measures of health, and they recommended that the UK government changed the measure used in their Levelling Up health goal from Healthy Life-Expectancy to a self-reported health measure.

Area level characteristics including deprivation have been shown to be associated with poor health independent of individual-level factors in many studies; see Oakes et al. (2015) for a recent review. Several different area-based measures of socio-economic position have been used in health research for England (Townsend et al. 1988; Carstairs and Morris 1991; Jarman 1983) including the IMD. These indices vary in the factors included and how they are weighted and combined (Allik et al. 2016). The most common are material deprivation, occupation, unemployment, education, and housing. The addition of the productivity and percentage of people living in towns components of the Stronger Town Index make our index somewhat different.

Research on migration and migration destinations within the UK suggests that health-selective migration changes the geographical distribution of poor health. Previous research using the ONS Longitudinal Study ('the LS') which comprises linked census records and life events data for approximately 1% of the population of England and Wales since 1971, found that between 1971 and 1991, inequalities in health increased between the least and most deprived areas, and that health selective migration, rather than changes in the deprivation of the area that non-migrants live in, accounted for the majority of change, and other research has shown relationships between migration and limiting long-term illness (LLTI) (Norman et al. 2005; Boyle et al. 2001; Wilding et al. 2016).

We chose to explore how general health was associated with our Stronger Towns Index. First, we looked at whether health in 2001 was associated with Town Strength, to justify its use as an index. We then looked at whether migration between 2001 and 2011 to an area of higher Town Strength was associated with good health in 2011 compared to migration to an area with lower Town Strength. Intuitively, we would expect healthier people to live in stronger places, because healthier people are attracted to stronger places, or because healthier people make places stronger, or stronger places make people healthier.

## Methods

To construct a Stronger Towns index for England’s Local Authorities, we gathered data on the five dimensions that had originally been proposed by MHCLG. We chose local authorities as they constituted a geographic area both for which data would be available and that would be able to administer funds. It was plausible that local authorities could include small parts of towns or more than one town entirely.

For productivity, we used sub-regional estimate using experimental Office for National Statistics (ONS) data on regional and sub-regional productivity. Data were taken from Table J4: Nominal Gross Valued Added GVA (B) (excluding rental income) per hour worked (at Local Authority level). A lower productivity led to a higher rank, indicative of greater need of funding). For income, we used estimates of gross disposable household income by local authority (ONS 2018). For skills, we used aggregate 2011 Census counts of the proportion of people with qualifications below GCSE C or equivalent (ONS 2013a). For deprivation, we used the IMD 2015 (MHCLG 2018), based on the proportion of Lower Super Output Areas (LSOAs) in the lowest decile, then the proportion in the second decile, etc. For the proportion living in towns, local authorities were ranked on the proportion of persons in the LA identified as living in small, medium, or large towns. Equally ranked LAs were ordered by population (ONS 2019). A higher proportion of people living in towns gave a higher rank.

The rank scores for each dimension were summed to give an (equally weighted) overall score. The overall score was then converted to a rank value, with the highest rank (1) representing the highest level of assumed eligibility. Local authorities were then grouped into rank deciles, and their aggregate characteristics were examined. We then matched up the 101 towns asked to apply for funds to the local authority in which they sit, and assigned their rank using our index.

To explore the association between Town Strength and health, a sample from England (the Towns Fund is not available in Wales) was drawn from the 2001 round of the ONS LS which comprises approximately 1.1% of the population of England and Wales, whose census records have been linked every 10 years between 1971 and 2011. The LS is representative of the whole population of England and Wales, including those in non-private households, has minimal bias due to non-response or attrition, high tracing rates (Lynch et al. 2015), and high response rates (ONS 2013b). Further information about the data can be found in the study’s Cohort Profile (Shelton et al. 2019).

Demographic and socioeconomic indicators of age and sex and highest educational qualifications in 2001 were included as potential covariates. LLTI in 2001 was also included in the analysis. Respondents were asked: ‘Do you have any long-term illness, health problem, or disability which limits your daily activities or the work you can do?’ with a note to ‘include problems which are due to old age’. For the dependent variable, we chose self-rated general health; respondents were asked: ‘Over the last 12 months would you say your health has on the whole been:...’ — the outcome measure was good health, compared with fairly good and not good combined.

The sample we used varied by analysis. The main sample (*n* = 407,878) was all members in England aged 16 and over in 2001 and whose records included a self-rated health response and a valid local authority code. LS members’ self-rated health outcomes were linked to their Stronger Towns Index rank decile by the local authority codes. We then derived a migration and social mobility (2001–2011) variable from these deciles. This had three categories: moved to an area within a lower decile of Town Strength, did not move or moved to area within the same decile for Town Strength, moved to an area within a higher decile of Town Strength.

Multivariate logistic regression was used to model the associations between self-rated health and Stronger Towns Index rank in a subsample of those who were also present in 2011, with information on highest educational qualification, migration, and sex.

## Results

### Town strength

**Fig. 1:**
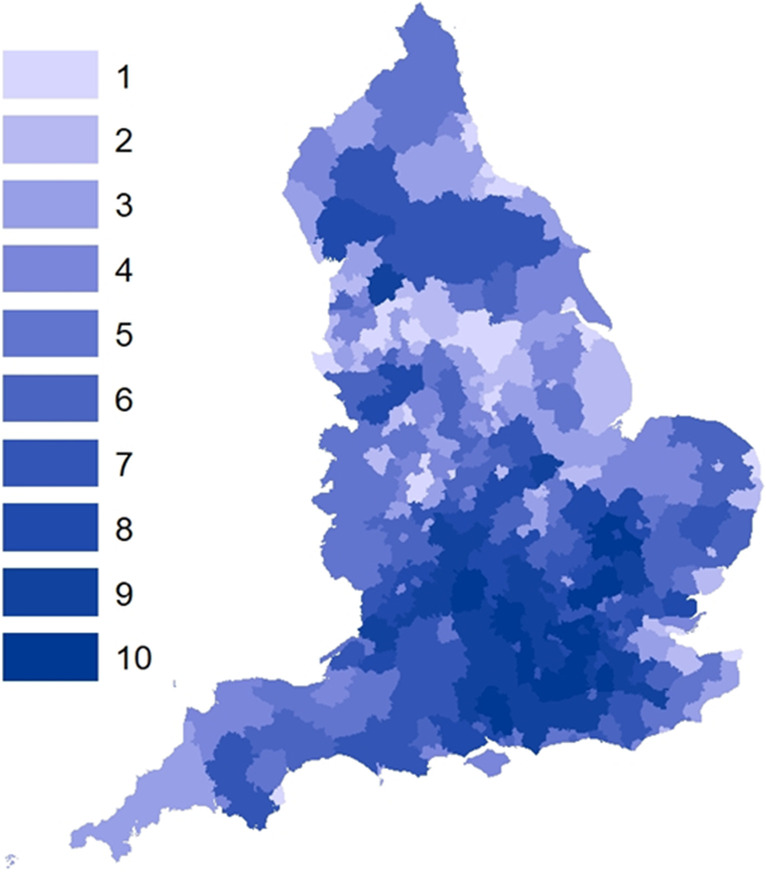
Local authority Stronger Town Index rank decile, England and Wales. Various data sources see paper. *Analysis authors’ own*

Table 1 shows the ten local authorities with the highest ranked ('weakest') index for Town Strength; these are mapped in Fig. 1. Local authorities in lower deciles tended to be in the economically deprived areas of the North of England, the Midlands and coastal areas. Five local authorities that contained towns that were selected to bid that did not receive funding from the first two tranches were ranked in the top 10 in our index. These were Redcar and Cleveland (Redcar), Doncaster (Doncaster and Stainforth), Walsall (Bloxwich and Walsall), County Durham (Bishop Auckland) and Rotherham. Table 2 shows the rank of the local authorities which are not in the top 100 in the index, in which there were towns which were flagged for funding. We identified 29 local authorities whose towns were invited to apply for funding, but which were not in our top 100 ranking local authorities. Within those 29, almost half (*n* = 14) have been awarded funding as of April 2021. We also identified 44 LAs that were in the highest 100 ranked in our index, but whose towns were not invited to apply for funding.

**Table 1:** The ten local authorities and their region with the highest ranked index for low Town Strength

Region	LA name	Rank
East Midlands	East Lindsey	1
North East	Redcar and Cleveland	2
Yorkshire and the Humber	Barnsley	3
West Midlands	Stoke-on-Trent	4
East Midlands	Bassetlaw	5
North West	Blackpool	6
Yorkshire and the Humber	Doncaster	7
West Midlands	Walsall	8
North East	County Durham	9
Yorkshire and the Humber	Rotherham	10

Data sources: MHCLG 2018; ONS 2013a; ONS 2013b; ONS 2018; ONS 2019

**Table 2:** Rank of the local authorities which are not in top 100 in the index, in which there are towns which are flagged to apply for funding

Region	LA name	Rank	Funded as of April 2021
East of England	Thurrock	107	
East Midlands	Lincoln	113	Y
Yorkshire and the Humber	Leeds	114	Y
West Midlands	Herefordshire, County of	120	
East Midlands	Erewash	122	
North East	Darlington	123	
West Midlands	Redditch	127	
South East	Hastings	136	
East of England	Norwich	144	
East of England	Ipswich	146	Y
East Midlands	Northampton	153	Y
North West	Warrington	158	
North West	Stockport	161	Y
West Midlands	Nuneaton and Bedworth	162	Y
East Midlands	Broxtowe	171	
South East	Lewes	175	
East of England	Colchester	179	Y
South West	Swindon	181	Y
North West	Preston	184	Y
East of England	Bedford	189	
West Midlands	Worcester	190	
East Midlands	Charnwood	208	
South West	Bournemouth	213	Y
North West	South Ribble	218	Y
East of England	Harlow	219	
East of England	Stevenage	224	Y
South East	Milton Keynes	230	Y
North West	Cheshire East	235	
South East	Crawley	267	Y

Data sources: MHCLG 2018; ONS 2013a; ONS 2013b; ONS 2018; ONS 2019

#### Association with health

**Fig. 2:**
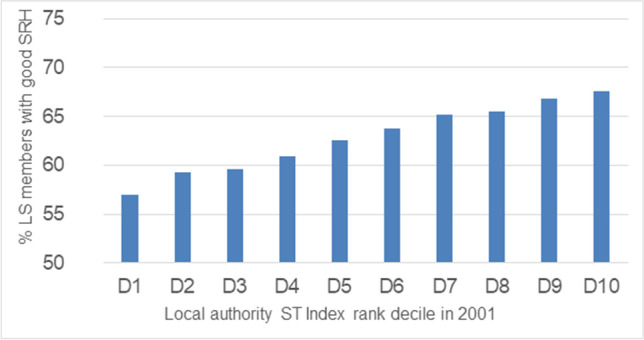
Proportion of LS members aged 16+ with good self-rated health (SRH) in 2001, by local authority Stronger Town (ST) Index rank decile in their residential location in 2001 (*N* = 407,878). Data Source various (see paper) including ONS LS. *Analysis authors’ own*

The proportion of adult LS members aged 16+ with good self-rated health in 2001 was 57% in Decile 1 with the lowest Town Strength, rising to 68% in Decile 10 with the highest Town Strength (Fig. 2). Bivariate analysis (not shown) revealed that significantly higher proportions of the LS members rated their health as good in higher deciles than in the lower deciles. It also revealed that there were significant differences in reporting good self-rated health by sex, age and education. Therefore, adjustment was made for these factors when modelling the association between self-rated health and decile by logistic regression. Table 3 shows the odds ratios for self-rated health - after adjustment for sex, age and education, LS members living in areas with higher deciles of Town Strength were significantly more likely (7% to 38%) to report good health than those in Decile 1.

**Table 3:** Odds of good self rated health in 2001 and assocation with Town Strength decile

	Odds ratio	*P*-value	Confidence interval	
Decile 1	1			
Decile 2	1.08	< 0.001	1.04	1.11
Decile 3	1.07	< 0.001	1.04	1.10
Decile 4	1.17	< 0.001	1.13	1.20
Decile 5	1.22	< 0.001	1.18	1.26
Decile 6	1.30	< 0.001	1.26	1.35
Decile 7	1.37	< 0.001	1.32	1.41
Decile 8	1.31	< 0.001	1.27	1.35
Decile 9	1.38	< 0.001	1.33	1.42
Decile 10	1.31	< 0.001	1.27	1.35

Adjusted for age, sex and highest educational qualification in 2001. Data sources: MHCLG 2018; ONS 2013a; ONS 2013b; ONS 2018; ONS 2019; and ONS Longitudinal Study. Analysis authors’ own. *N* = 368,507

#### Assocation with mobility

Table 4 shows a matrix of the proportion of people in each decile in 2001, by decile in 2011. The vast majority of people were resident in the same decile in 2011 as they were in 2001. The highest proportion of ‘stayers’ was in Decile 1 (the lowest decile for town strength). Bivariate probability analysis (not shown) revealed significant differences in the proportions of LS members reporting good self-rated health by change in decile; the lowest proportion among those who remained in Decile 1 and the highest among those who were in Decile 10 in 2011. We therefore looked at the association between good self-rated health and any positive change between decile compared to any negative change in deciles between 2001 and 2011. Moving to an area with a higher decile for Town Strength was not associated with higher odds of good self-rated health in 2011 when compared to moving to an area with lower Town Strength after adjustment for age, sex, and highest educational qualifications (OR=1.04, 95%CI [0.99-1.09]. Remaining in the same decile was however associated with lower odds of good self-rated health (OR=0.93, 95% CI [0.89, 0.96], n=299,008).

**Table 4:** Local authority Stronger Towns Index rank decile migration 2001 to 2011

		1	2	3	4	5	6	7	8	9	10
Local authority ST Index rank decile in 2011	1	**90.3**	2.4	2.1	2.8	1.3	1.4	0.9	0.7	0.6	0.5
	2	1.9	**85.9**	1.8	2.5	2.0	2.4	1.5	1.75	0.7	0.8
	3	2.2	2.3	**86.8**	2.2	1.95	1.9	2.8	1.6	1.1	1.2
	4	1.7	2.1	1.6	**82.2**	2.0	2.5	1.7	1.6	0.8	1.0
	5	1.0	1.7	1.5	2.1	**81.9**	2.6	2.4	1.9	1.8	1.5
	6	0.8	1.6	1.3	2.1	2.2	**79.5**	2.2	2.5	1.6	1.2
	7	0.6	1.2	1.7	1.7	2.4	2.7	**78.2**	2.3	3.2	2.3
	8	0.55	1.3	1.15	1.8	2.1	2.8	3.0	**79.5**	3.7	3.8
	9	0.5	0.8	1.1	1.3	2.25	2.4	3.8	3.9	**79.7**	5.5
	10	0.4	0.65	0.9	1.3	1.8	1.9	3.5	4.3	6.8	**82.3**
	Local authority ST Index rank decile in 2011										

Figures in bold font represent non-migration or migration within same decile. Data sources: MHCLG 2018; ONS 2013a; ONS 2013b; ONS 2018; ONS 2019; and ONS Longitudinal Study. Analysis authors’ own

## Discussion

Our Stronger Towns Index was built at local authority level using openly available versions of the original indicators or proxies thereof that were proposed by MCHLG. The geography of Town Strength was similar to other deprivation geographies, with high levels of deprivation in the North West and lower levels in the South East. By adding the proportion of people living in towns, it demonstrates that there is poverty beyond those regions. The MCHLG choice of towns does not capture the same areas as our index. Notably, our index identifies the Midlands as potentially missing out on funds. Also Swale in the South East did not get selected to apply for funding by 2021, yet scored very highly in our index and was the focus of media attention in late 2020 for having the second highest rate of COVID-19 infection (BBC 2020). Of the areas that were selected to apply for funding, half of those we ranked in the top ten for low Town Strength did not receive funding in the first two tranches.

People living in economically stronger areas, as indicated by Stronger Town Index rank decile, were significantly more likely to rate their health as good, even after accounting for factors known to be associated with self-rated health. Research has shown that migration is less frequent among those in poor health (Wilding et al. 2016). This extends research on the healthy migrant effect, but in relation to other migrants rather than just those who do not move. It was non-migration combined with remaining in similar places that was associated with lower odds of good health, perhaps demonstrating the resilience education provides for those who move to ‘weaker’ areas. This is of current policy interest, with national relocation of those in social housing from overcrowded locations, e.g. London to lower income areas.

## Data Availability

The results are derived from the ONS Longitudinal Study, which is only available through ONS Secure Research Service. The component records cannot be re-disseminated.
